# How to strengthen a health research system: WHO’s review, whose literature and who is providing leadership?

**DOI:** 10.1186/s12961-020-00581-1

**Published:** 2020-06-23

**Authors:** Stephen R. Hanney, Lucy Kanya, Subhash Pokhrel, Teresa H. Jones, Annette Boaz

**Affiliations:** 1 Health Economics Research Group, Institute of Health, Environment and Societies, Brunel University London, Uxbridge, UB8 3PH United Kingdom; 2 Department of Health Policy, London School of Economics and Political Science, London, United Kingdom; 3 Faculty of Health, Social Care and Education, a partnership between Kingston University and St George’s, University of London, London, United Kingdom

**Keywords:** Biomedical research, Capacity-building, Evidence-based practice, Health ministries, Health research systems, Health services research, Policy-making, Priority-setting, Research utilisation, Sustainable Development Goals, Translational medical research

## Abstract

**Background:**

Health research is important for the achievement of the Sustainable Development Goals. However, there are many challenges facing health research, including securing sufficient funds, building capacity, producing research findings and using both local and global evidence, and avoiding waste. A WHO initiative addressed these challenges by developing a conceptual framework with four functions to guide the development of national health research systems. Despite some progress, more is needed before health research systems can meet their full potential of improving health systems. The WHO Regional Office for Europe commissioned an evidence synthesis of the systems-level literature. This Opinion piece considers its findings before reflecting on the vast additional literature available on the range of specific health research system functions related to the various challenges. Finally, it considers who should lead research system strengthening.

**Main text:**

The evidence synthesis identifies two main approaches for strengthening national health research systems, namely implementing comprehensive and coherent strategies and participation in partnerships. The literature describing these approaches at the systems level also provides data on ways to strengthen each of the four functions of governance, securing financing, capacity-building, and production and use of research. Countries effectively implementing strategies include England, Ireland and Rwanda, whereas West Africa experienced effective partnerships. Recommended policy approaches for system strengthening are context specific. The vast literature on each function and the ever-growing evidence-base are illustrated by considering papers in just one key journal, *Health Research Policy and Systems,* and analysing the contribution of two national studies. A review of the functions of the Iranian system identifies over 200 relevant and mostly national records; an analysis of the creation of the English National Institute for Health Research describes the key leadership role played by the health department. Furthermore, WHO is playing leadership roles in helping coordinate partnerships within and across health research systems that have been attempting to tackle the COVID-19 crisis.

**Conclusions:**

The evidence synthesis provides a firm basis for decision-making by policy-makers and research leaders looking to strengthen national health research systems within their own national context. It identifies five crucial policy approaches — conducting situation analysis, sustaining a comprehensive strategy, engaging stakeholders, evaluating impacts on health systems, and partnership participation. The vast and ever-growing additional literature could provide further perspectives, including on crucial leadership roles for health ministries.

## Background

Interest in strengthening health research systems has intensified following increasing recognition of the importance of research in achieving key goals such as universal health coverage [1] and the Sustainable Development Goals (SDGs) [2]. However, achieving progress in health research faces many challenges, including securing sufficient funds [3–9], building and retaining capacity [3, 7, 10–14], producing research findings, and using both local and global evidence [1, 15–20].

Chalmers and Glasziou [21] dramatically highlighted the extent of the challenges facing health research by claiming, in 2009, that even where there was funding and capacity, up to 85% of all biomedical research was wasted because it asked the wrong questions, was poorly designed, or was either not published or poorly reported, with only about 50% of studies being published in full.

Many of these challenges have long been recognised and the adoption of a systems approach advocated. In 2000, the Bangkok Declaration on Health Research for Development promoted the importance of a systems approach, following consideration of how a health research system could *“be integrated with a nation’s health development plan”* [15]. It suggested that establishing and strengthening an effective health research system needed coherent and coordinated health research strategies [15]. National strategies should have specific combinations of various health research system components, tailored to the country’s circumstances.

The WHO’s Knowledge for Better Health initiative involved further work on these issues [3, 16]. The Mexico Statement on Health Research, issued in 2004 by a Ministerial Summit, called for nations to take actions to strengthen their national health research systems (NHRSs). It was endorsed in 2005 by the Fifty-eighth World Health Assembly in a resolution committing its Member States to strengthening their NHRSs as a pathway to improve their overall health system [22].

As part of the initiative, Pang et al. [3] developed a conceptual framework to guide the analysis and strengthening of health research systems, including development of a health research strategy. While this can be used for planning, monitoring and evaluation of health research systems, it did not claim to provide a precise blueprint. The framework defined a health research system as *“the people, institutions, and activities whose primary purpose in relation to research is to generate high-quality knowledge that can be used to promote, restore, and/or maintain the health status of populations; it should include the mechanisms adopted to encourage the utilization of research”* [3].

The framework indicates the range of constituent components and how they can best be brought together into a coherent system. It identified four main functions for an effective system, namely stewardship, financing, capacity-building (or creating and sustaining resources), and producing and using research [3]. Each function is defined by operational components and consists of one or more of a total of nine such components.

Since then, progress is evidenced by analyses of developments in individual countries, including the National Institute for Health Research (NIHR) in England [23–25], and in repeat surveys conducted in various WHO regions, including Africa [4, 26, 27] and the Pan-American Health Organization (PAHO) [28]. However, as reported by those surveys and other publications, many challenges remain. For example, in February 2020 a new analysis by the WHO Global Observatory on Health R&D examined health research funding, concluding that *“neglected diseases such as those on the WHO list of neglected tropical diseases remain very neglected in terms of R&D investments”* [29].

Nevertheless, there are various initiatives underway, including in WHO’s Regional Office for Europe, which commissioned an evidence synthesis on the topic as part of its Action Plan to Strengthen the use of Evidence, Information and Research for Policy-making in the WHO European Region [18]. The synthesis is published in the WHO Region’s Health Evidence Network (HEN) report series and consists of a scoping review addressing the question *“What is the evidence on policies, interventions and tools for establishing and/or strengthening NHRSs and their effectiveness?”* [30].

The evidence synthesis focuses on the systems level and so primarily includes publications taking a systems approach at either the national or multi-national level. Not surprisingly, *Health Research Policy and Systems* (*HARPS*) is the single largest source of papers included in the HEN report. These were papers directly identified in the review’s search or papers included in the HEN report to illustrate a key point because they had been cited in one of the WHO reports or other systems-level collations of papers included in the synthesis.

While the system level papers did provide considerable data about each function, limited resources to conduct the scoping review meant that we had to exclude papers focusing solely on one specific function of a health research system or on just one field of health research. As acknowledged in the HEN report’s agenda for further research, there is a large number of publications (papers and grey literature) covering each function [30]. Therefore, reviewing all of these publications would be a major task but some exploration of the extent of the task, and the nature of such literature, could be informative. Furthermore, additional papers are continuously emerging, including from the various initiatives that are ongoing or just underway, for example, the European Health Research Network [31].

The three sections of this paper sequentially address the question of how to strengthen a health research system by:

1. Describing key points and conclusions from WHO’s HEN report.
2. Illustrating the nature of the ever-widening literature available on each function, or component, of a health research system by examining two sources in particular. First, the full range of papers published in *HARPS* in the 30 months up to February 2020. Second, the range of data gathered from publications or interviews that is included in detailed studies of the national health research systems in two countries – Iran [32] and England [33]; between them, these two papers also illustrate diverse aspects of the additional material that could be drawn upon.
3. Considering a key question in the analysis of the current and future initiatives, namely who is going to steer the development of health research systems? Here, information and insights from the HEN about this sometimes-controversial issue, along with wider continuing analysis, are drawn on in the more flexible and speculative way that can be undertaken in an Opinion piece compared to a formal evidence synthesis.

## WHO’s review, whose literature and who is providing leadership?

### WHO’s review

The evidence synthesis described by the HEN report [30] starts by describing the importance of NHRSs in helping to achieve universal health coverage [1] and the SDGs [2]. It goes on to analyse the challenges facing health research and describes how issues remain unresolved despite the development and application of a systems approach including WHO’s framework for health research systems [3]. Many countries do not have comprehensive national health research policies or strategies that would facilitate the introduction of a systems approach. Therefore, challenges remain around two key and overlapping sets of issues. First, how to develop a systems approach to maximise the benefits from the research resources available – this can be a challenge even in high-income countries with considerable research funding. Second, how best to strengthen each specific function and component of a health research system [30].

The HEN identifies two main systems-level approaches to strengthening NHRSs. The first is comprehensive and coherent strategies, which can be contained in either policy documents, such as those from the English NIHR [34], the Irish Health Research Board (HRB) [35] and the Rwandan Ministry of Health [14], or in specific legislation as in the Philippines [36]. The second systems-level approach involves partnerships and multi-country initiatives, especially with international organisations. Two initiatives from the West African Health Organization (WAHO) are particularly important examples [5, 37]. Here, the ministries of health of the 15 West African member countries worked together in a joint initiative covering all the countries and with funding and expertise from a range of partners, including the Council on Health Research for Development (COHRED), the Canadian International Research Centre, the Special Programme for Research and Training in Tropical Diseases, and the Wellcome Trust. All WHO Regions have seen multi-country activities by WHO and/or COHRED to strengthen NHRSs, including the repeat surveys that identify areas for action [4, 26, 28].

Then, broadly using the WHO framework as the structure [3], the HEN identifies key points from systems-level literature on each of the four functions and nine components. The components of the stewardship and governance function include defining a vision, ethical review, research priority-setting, and appropriate monitoring and evaluation [3]. Consultation with health system stakeholders should enhance the relevance of the research priorities to the healthcare system, with examples of extensive priority-setting engagement activities sometimes being seen as a key aspect of building the NHRS as in Brazil [38]. Evaluating the impact of research on policy and practice should help researchers to focus on achieving such impact and was therefore promoted in the World Health Report 2013 [1].

Securing finance can involve obtaining funding from sources within the country and from external donors or multi-national organisations [30]. Targets for research expenditure, such as the 2% of national health expenditure set by the 1990 Commission on Health Research for Development [39], can usefully be brought into health research system strategies as in Rwanda [14]. Major health research strategies from countries within the European Union can highlight the importance of European Union funding as in France [40], Ireland [35] and Malta [41]. Requests for funding can be more effective when linked to other parts of the overall strategy, including identified priorities that need supporting through donor funding [42] and assessments of the benefits obtained from previous funding such as in England [24].

Capacity-building involves building, strengthening and sustaining the human and physical capacity to conduct, absorb and utilise health research [3]. In 2016, Santoro et al. [43] identified the generally low levels of research production in 17 countries of the former Soviet Union and south-eastern Europe and made recommendations for the sustained investment in training and career development of researchers, which should go beyond scholarships for training abroad and involve comprehensive strategies to ensure clear career structures. Strategies such as that from Inserm in France set out comprehensive plans for capacity-building [40] and strategies in both England and South Africa addressed priority gaps identified in the research capacity within the healthcare professions [34, 44]. Donors can play an important part in building capacity but, recognising the need to avoid donor domination, often do so through partnerships. These can take diverse forms ranging from multi-country initiatives, such as that by WAHO, which included an initiative focusing on the challenges of post-conflict countries but was unable to meet all the needs [37], to accounts that focus on the partnership to address a broad range of capacity issues in a single country such as Malawi [7], to partnerships between individual institutions. Examples of the latter can feature particular challenges – the James Cook University in Australia worked with the Atoifi Adventist Hospital in Malaita, the most populous province of the Solomon Islands, to start establishing health research system capacity on the island using an inclusive, participatory approach [45]. Increasingly, there are also south–south partnerships, for example, an account of the Panamanian health research system described how the country’s first doctoral programme in biotechnology was established with support from Acharya Nagarjuna University in India [46]. The Rwandan strategy described plans to tackle the ‘brain drain’ through making the country an appealing place to conduct health research in terms of job requirements and providing opportunities for career advancement [14].

The three mutually reinforcing components of the producing and using research function encourage the production of scientifically valid findings that are relevant for users and communicated to them in an effective manner [30]. Major research funding bodies increasingly seek to address the waste issues raised by Chalmers and Glasziou [21] by working together in the Ensuring Value in Research (EViR) Funders’ Collaboration and Development Forum. It issued a consensus statement committing the organisations signing it to *“require robust research design, conduct and analysis”* [47]. The Forum is convened by the English NIHR, the Netherlands Organization for Health Research and Development, and the Patient-Centered Outcomes Research Institute (United States) with the active support of major research funding organisations from Australia, Ireland (HRB), Italy, Sweden and Wales, plus the Special Programme for Research and Training in Tropical Diseases [48]. The first WAHO intervention also worked to boost research publications, including by creating a regional peer-reviewed, multilingual journal [5]. How research is produced can increase the chance that the evidence will be used in the health system, for example, the English NIHR strategy noted that leading medical centres with substantial funding to conduct translational research can act as “early adopters of new insights in technologies, techniques and treatments for improving health” [34].

Fostering the use of research requires specific knowledge translation and management approaches that draw on both locally produced and globally available evidence. Various health research strategies promote the role of Cochrane, including in England, where a unified knowledge management system to meet the needs of various stakeholders, including patients and their carers, involves funding both Cochrane and a review centre focusing on the needs of the National Health System [34]. In Ireland, the HRB strategy facilitated evidence-informed decisions through promoting access to the Cochrane Library and supporting training in conducting high-quality Cochrane reviews [35]. South Africa Cochrane featured as an important element in the NHRS [44]. The Rwandan strategy stated that *“The Government of Rwanda is committed to using research findings to make evidence-based decisions that will improve health in Rwanda”* [14]. It aimed to orientate various functions, including agenda-setting, monitoring and evaluation, and capacity-building, towards facilitating this challenging aim. The World Health Report 2013 highlighted various mechanisms that health research systems could adopt, including EVIPNet (Evidence-informed Policy Network), to promote the use of research [1, 49].

The review also considers the effectiveness of approaches to strengthening NHRSs. Several reviews identified the effectiveness of the comprehensive approach taken by Professor Dame Sally Davies in creating the English NHRS [23, 25, 50]. The title of one analysis, ‘NIHR at 10: 100 examples, 10 themes, 1 transformation’, emphasises that the success of the NIHR depended on a range of elements being brought together in one transformation [25, 50]. One of the 10 themes was the involvement of patients in decisions about research priorities and processes and, based on this, another recent analysis highlighted England and Alberta (Canada) as having health research systems that had made important progress [51]. Davies herself reflected on the success of the NIHR and stated: *“What we envisaged was integrating a health research system into the health care delivery system so that the two would become interdependent and synergistic*” [24]. WHO’s Regional Office for Africa drew on their series of surveys of the performance of countries in building NHRSs and analysed the data from the 2014 and 2018 surveys using the NHRS barometer that they developed to score progress on a range of items linked to the list of NHRS functions [11, 26]. In the 2014 survey, the Rwandan system was identified as the best performing and it, along with the majority of systems, was reported to have further improved in the 2018 survey; by then, South Africa was reported to have the best performance in Africa. The surveys also illustrate how the multi-country approach makes a useful contribution to strengthening NHRSs by helping to target action. Furthermore, the WAHO interventions made some progress but, while the evaluations identified the importance of political will and leadership provided by WAHO’s parent organisation of West African states, they also emphasised that building capacity for a whole NHRS is a significant task requiring commitment over the long-term [17, 37].

The HEN review collated a range of examples of tools for NHRS strengthening. These were identified from the systems level discussions of NHRS strategies and partnerships and/or the major reports calling for NHRS strengthening such as the World Health Report 2013 [1]. The HEN lists these in an Annex [30].

The discussion in the HEN draws on the literature that was included to identify five key policies that those responsible for strengthening NHRSs could consider [30], namely conduct context, or situational, analyses to inform strengthening activities [5, 34, 35, 37, 52–54], develop a comprehensive and coherent strategy [14, 34–36], engage stakeholders in the development and operation of the strategy [7, 23, 34, 35, 38, 41, 44, 51, 55–59], adopt monitoring and evaluation tools that focus on the objectives of the NHRS, including health improvement [1, 14, 24, 60, 61], and develop partnerships [5, 11, 28, 37, 62]. Examples of the evidence to support or illustrate each policy are given in Table 1.

**Table 1:** Policies to strengthen National Health Research Systems and supporting evidence

Policy to strengthen National Health Research Systems	Examples of evidence supporting or illustrating each policy (selected from the body of evidence presented in the HEN [30])
Conduct context or situational analyses of current national position to inform strengthening activities	COHRED, in particular, has developed tools to assist countries in conducting situational analyses as part of wider advice [52] and this approach was an important element in the WAHO interventions being successful to the extent that they were [5, 37, 53, 54]. Strategies informed by analyses of their current situation include those for the English NIHR [34] and the Irish HRB [35]
Develop a comprehensive and coherent NHRS strategy	Comprehensive and coherent strategies with at least some degree of success (as seen in progress on some or all of the NIHR functions) had set out how they intended to take action on the range of health research system functions and components, even if not necessarily explicitly using the WHO framework [3]; examples include the strategies for the English NIHR [34], the Irish HRB [35], and in the Philippines [36] and Rwanda [14]
Engage stakeholders in the development and operation of the NHRS strategy	Strategy documents such as those for the NIHR [34] and HRB [35], plus ones in British Columbia [55], Malta [41] and New Zealand [56], describe the importance and/or range of stakeholders engaged in developing the strategy. Articles describing the approach in South Africa [44] and Zambia [57] also highlighted the importance of wide stakeholder engagement. An analysis of stakeholder engagement in the creation and operation of the NIHR identified it as making a key contribution to its success [23]. There is increasing support for the engagement of stakeholders in setting the priorities for research as well as in research processes and translation [7, 38, 51, 58, 59]
Adopt monitoring and evaluation tools that focus on the objectives of the NHRS, including health system improvement	A range of documents, including ones on the NIHR [24], HRB [60] and Rwandan strategies [14], and the World Health Report 2013 [1], demonstrate the importance of adopting monitoring and evaluation approaches that include a focus on assessing the impacts of research on health polices/practice and the economy, e.g. through application of the Payback Framework [60, 61]
Develop/participate in partnerships across regions, bilaterally or within the NHRS	Examples of progress made by partnerships between countries, sometimes along with international organisations and donors, include the WAHO interventions [5, 37, 53, 54] and the work of WHO regional offices for Africa [11, 26] and the PAHO [28, 62]

Source: Data extracted from Health Evidence Network report 69 (Hanney et al., 2020) [30]

*COHRED* Council on Health Research for Development, *HEN* Health Evidence Network, *HRB* Health Research Board, *NHRS* National Health Research System, *NIHR* National Institute for Health Research, *PAHO* Pan-American Health Organization, *WAHO* West African Health Organization

In summary, therefore, this section shows that the WHO evidence synthesis, published as a HEN report [30], provides a firm basis for decision-making by policy-makers and research leaders looking to strengthen the health research system in their country. It analyses, in turn, the individual functions and components within a system and identifies a series of tools that can be used for strengthening many of them. Finally, this section highlights the five crucial policy approaches that the HEN report suggests can be applied as appropriate to the context of the country (Table 1).

### Whose literature?

As noted above, the HEN was a scoping review and focused on the literature at the systems level rather than on publications (papers and grey literature) related solely to specific functions, types or fields of research [30]. Therefore, there is scope for further work to incorporate an even wider range of publications than the 112 included in the HEN review [30]. The discussion in the HEN suggests that further research could usefully take the form of a series of reviews on the extensive literature on each of the NHRS functions or components, which could then be collated [30]. Just two of the many available sources illustrate the nature of the vast literature available on each function, or component, of a health research system and the way the literature on that, and the system level developments, is ever-widening. First, we can examine the papers published in *HARPS,* the specialist journal in the field of building NHRSs. Second, we can focus on two very different but detailed studies of individual NHRSs – one conducted for a PhD thesis to show the 50 year history of the development of all the functions in the Iranian health research system [32] and the other an interview-based study to understand the factors behind the creation of the NIHR with its new strategy [33].

In terms of further reviews of the literature on specific functions or components, *HARPS* would probably be a key source. In the summer of 2017, an analysis by the retiring editors of the papers published in the journal from its inception in 2002 identified many papers that had been published on each of the functions or components of a health research system [63]. While this editors’ analysis was included in the HEN review because it organised its discussion of the papers at the systems level, the individual papers in it were, in general, only included in the HEN review if they, too, adopted a systems approach at the national or partnership level, or were also cited in a report such as the World Health Report 2013 [1]. Examples of such papers include Viergever et al. on priority-setting [59], Bates et al. on capacity-building [64], and Lavis et al. on the SUPPORT tools for evidence-informed policy-making [65]. Therefore, many additional papers related to specific functions (or fields) could be consulted, in a formal review or otherwise, in any future series of reviews, each with a narrow focus on strengthening a specific function.

To further inform this current Opinion piece, a quick ‘hand-search’ was conducted of the papers published in *HARPS* in the 30 months since the previous analysis in mid-2017 [63]. This again identified a wide range of papers on specific components, especially priority-setting, evaluation of research impacts, capacity-building and the translation of research (or knowledge mobilisation). Various papers linked the final two points and discussed capacity-building and knowledge translation [13, 66]. Such a focus is entirely consistent with the aim described by the incoming editors in Autumn 2017 of bringing *“all elements of the research–policy world together – such that the research which is done is useful and that it is used”* [67]. In this more recent phase of *HARPS*, there have also been important papers on issues related to the policies ‘recommended’ at the end of the HEN and listed above, including the contribution of stakeholder engagement in research [68].

The more recent papers could sometimes provide useful further tools on specific functions. Their narrow focus meant they had not been directly included through the HEN search and, further, they had not been included in any of the major reports also used as sources for tools such as the World Health Report 2013 [1]. In some instances, this was because they were too recent, for example, the ISRIA statement by Adam et al. [69] describing the ten-point guidelines for an effective process of research impact assessment prepared by the International School on Research Impact Assessment (ISRIA). Even more recently, the Intervention Scalability Assessment Tool, developed by Milat et al. [70], was proposed for use not only by health policy-makers and practitioners for selecting interventions to scale up but also to help design research to fill evidence gaps. This analysis of the papers from just one journal reinforces the message that there is likely to be a plentiful supply of literature for a future review on any of the main specific components.

This message is further reinforced by a more detailed analysis of the papers in *HARPS* in the first 2 months of 2020. Articles on the main components of a NHRS were supplemented by some important papers on topics that are highly relevant but which feature less frequently in *HARPS.* These include a study aimed at reducing the research waste that arises from disproportionate regulation by examining the practices for exempting low-risk research from ethics review in four high-income countries [71], the Global Observatory’s paper on research funding described earlier [29], a study on the governance of national health research funding institutions [72], and one on a more recent topic of growing significance – an analysis of attempts to boost gender equality in health research [73]. Additionally, some of the papers on specific components, such as impact evaluation or use of evidence, are extending the analysis. Examples include consideration of how research impact assessments are implemented in practice within research organisations [74] and how evidence is used in decision-making in crisis zones [75]. To illustrate the volume of studies being produced, there has been a flurry of studies, in the first 2 months of 2020 alone, on the collaboration and coproduction of health research. The titles include ‘Building an integrated knowledge translation (IKT) evidence base: colloquium proceedings and research direction’ [76], ‘Using a ‘rich picture’ to facilitate systems thinking in research coproduction’ [77], ‘Exploring the evolution of engagement between academic public health researchers and decision-makers: from initiation to dissolution’ [78], ‘Research co-design in health: a rapid overview of reviews’ [79], and ‘Conceptualising the initiation of researcher and research user partnerships: a meta-narrative review’ [80].

Finally, another article in May 2020 presented a new conceptual model for health research systems to strengthen health inequalities research [81]. Here, we have focused on just one journal, *HARPS,* because it was the largest single source of papers in the HEN report, which totalled 140 publications (additional publications were included to the 112 in the review to help set the background, provide examples of key tools, etc). However, even with the review’s focus on the system level, *HARPS* only provided 22% (31 out of 140) of the publications; 31% (43 of 140) came from other journals and 47% (66 of 140) were other types of publication. If the focus was shifted to including papers on specific functions it is highly likely that there would be a higher proportion of papers from other journals.

The authors of two single-country papers on the development of the health research system, Mansoori [32] about Iran and Atkinson et al. [33] on the creation of the NIHR in England, both highlight the importance of context but also claim their findings could have wider application. Examining these two papers is also informative because of the differences between the studies, including one being located in a low- or middle-income country, and the other not.

Mansoori’s narrative review of studies addressing the health research system of Iran included 204 relevant and mostly national records, categorised using an approach informed by the functions and components of WHO’s NHRS framework [32]. The papers and grey literature documents included were all available in English or Persian, and mostly published in journals other than *HARPS,* and illustrate the vast literature available at a global level on the various components of a NHRS*.* They informed an impressively detailed account of the various NHRS components and the attempts to strengthen them. For example, the account of the development of the national level ethical overview includes a fully documented chronology of the progress over 25 years and some insightful analysis of how the progress was facilitated by the pivotal role of Professor Bagher Larijani, who was a prominent medical practitioner, leading researcher and founder of the Medical Ethics Research Centre in Iran. He was able to *“use the confidence that Iranian authorities had in him as an opportunity”* [32].

While Mansoori’s review was included in the HEN review, only a tiny fraction of the available data about Iran could be included, primarily in a brief description of the system’s effectiveness [30]. However, the full paper could usefully inform the approach of researchers and/or policy-makers planning a detailed analysis of their own NHRS prior to embarking on exercises to strengthen it, and *“*[t]*he findings emphasized that improvement of HRS functions requires addressing context-specific problems”* [32]. As an illustration, Mansoori’s review identified a need for “*a more systematic, inclusive”* approach to research priority-setting [32] and, in the same stream of research, she co-led just such a priority-setting exercise to help address the knowledge gaps related to achieving both Iran’s national health policies and the SDGs [82].

Atkinson et al. examined the creation of what might be viewed as the most successful attempt to strengthen a health research system in their paper ‘‘All the stars were aligned’? The origins of England’s National Institute for Health Research’ [33]. Compared with Mansoori, the authors adopted a different but equally detailed approach in their analysis, which was conducted principally through interviews and a witness seminar but also drew on the existing literature and documents [33]. They showed how the formation of the NIHR was led from the Department of Health by a key group driven by Sally Davies. They aimed to improve patient care through both the strengthening of evidence-based medicine and through boosting the infrastructure to facilitate pharmaceutical clinical trials that would also meet wider industrial and economic goals.

As with Mansoori’s study, consideration was given to how the full analysis could be informative to any planned detailed study or reforms in any other country. The key observations were similar to the recommendations from the HEN report with a focus on stakeholder engagement and building support: *“*[t]*wo measures likely to contribute to political support are to place the greatest emphasis on ‘problem’ rather than ‘investigation’ research, and to devote attention to measuring and reporting research ‘payback’*” [33]. Atkinson et al.’s paper is also a link to the other main source considered here because it was a recent paper published in *HARPS.*

In summary, if further analysis and research beyond that in the WHO evidence synthesis [30] is thought to be relevant in the particular country looking to strengthen its health research system, this Opinion piece indicates some of the types of additional sources of information that are available and how they might be organised. The vast literature on each function and the ever-growing evidence base are illustrated by considering papers in just one key journal, *HARPs,* and analysing the contribution of two national studies. A review of the functions of the Iranian system identifies over 200 relevant, mostly national, records and an analysis of the creation of the English NIHR describes the key leadership role played from the health department.

### Who is providing leadership?

The above analysis demonstrates that there is no shortage of useful material on which to draw when strengthening health research systems. However, key questions remain as to who might best lead or steer attempts to strengthen such a system.

The papers by both Mansoori [32] and Atkinson et al. [33] illustrate that, where a key committed individual has the capacity and opportunity to provide leadership, this can be a vital element in making progress. However, the institutional factors are also crucial.

The HEN developed the argument that a department or ministry of health will have a particular interest and perhaps experience in promoting research agendas that meet the needs of the healthcare system and in helping to develop mechanisms to use the findings from such research, where appropriate, to inform local policy and practice [30]. The health ministry or a research council responsible to it played an important role in the various systems identified above as being effective, as was also the case in the WAHO initiative [30]. In some cases, as with Zambia, more progress was made once the ministry of health elected to play a more important role, sometimes in place of other stakeholders [57]. Examples of the important role that health ministries can play were described in the 2013 World Health Report, including on Paraguay: “*the support of the Minister of Health backed by the President of Paraguay has been a key factor in the development of a national health research system”* [1]. Additionally, naturally enough, the activities of the various WHO regional offices in boosting NHRSs tend to focus on working with the national ministries of health, including work in Europe [31] and by PAHO [28]. Conversely, several analyses illustrate that progress in strengthening the NHRS might be limited where key parts of the ministry of health, for whatever reason, do not provide support [9, 83].

Nevertheless, some disadvantages or dangers were identified when the ministry of health plays the leading role. First, in England prior to the creation of the NIHR as well as in some other countries, the research funds controlled by the health ministry were sometimes appropriated by other parts of the health system when they were under particular pressure for resources [84]. Similarly, there have been a few reports that health research funding lost out when donor funds that had previously been allocated specifically for health research programmes were replaced by donations of funds to be allocated by the nation’s own health system according to its own priorities [85, 86]. One way of attempting to mitigate the danger is, as undertaken by the NIHR and described by Atkinson, by building support for health research through measuring and reporting the payback from research [24, 33].

The second danger arises because, traditionally, many researchers argued that the best science came when they had the freedom to identify the key research topics, rather than having priorities set by others [84]. Therefore, they argued, the responsibility for funding and organising health research should be left to organisations that are part of the research system and independent of the health system [84]. Furthermore, despite the growth of interest in coproduction approaches noted above, there have also been recent doubts raised about the assumption that coproduction is always the most appropriate approach [87]. This issue clearly requires sensitive handling. Indeed, Atkinson et al. [33] argue that one of the great successes of the NIHR is that this issue has been so skilfully handled by the NIHR that external input, or stakeholder engagement, in setting agendas has become widely accepted and the structures created give ministers a sense of ownership without sacrificing scientific independence.

The efforts of WAHO [5, 37] and the WHO regional offices for Africa and PAHO [11, 26, 28, 62] indicate that partnerships can be helpful. In Europe, the WHO regional office worked with Member States to create the European Health Research Network, which is intended to help nations with limited NHRSs who wish to make more progress [31].

Partnerships can provide important support and encouragement, but the evidence suggests there must be strong political will somewhere within the political and/or health systems for a health research system to be fully strengthened. The Central Asian countries in WHO’s European Region seem to provide an illustration of this point. A COHRED collaborative initiative successfully resulted in situation analyses being produced in each country and then jointly discussed as the basis for action [88], but according to the analysis by Santoro et al. [43], limited progress seems to have been made in the subsequent years.

The importance of partnerships and collaboration in focusing research efforts in an extreme crisis, with a leadership role for the WHO, has been seen in the race to find treatments for COVID-19 and vaccines against severe acute respiratory syndrome coronavirus 2 (SARS-CoV-2), which causes the COVID-19 disease [89]. In many NHRSs across the globe, including in the Philippines, scientists are coming together to participate in WHO’s Solidarity Trial, which will test the safety and effectiveness of various possible therapies for treating COVID-19 [90]. Sarah Gilbert, leader of Oxford University’s Jenner Institute’s work on developing one of the leading vaccine candidates explained that cooperation was vital for tackling the crisis: *“Work is continuing at a very fast pace, and I am in no doubt that we will see an unprecedented spirit of collaboration and cooperation, convened by WHO, as we move towards a shared global goal of COVID-19 prevention through vaccination”* [91]. A key issue going forward is how such cooperation can be built on in strengthening NHRSs into the future. For now, it is recommended that a prospective study be conducted to analyse all that is being done in different NHRSs to speed up research during the pandemic, with a view to taking lessons about cooperation, partnerships and other matters into strengthening NHRSs in the future [89].

## Conclusions

The WHO evidence synthesis, published as a HEN report [30], provides a firm basis for decision-making by policy-makers and research leaders looking to strengthen the health research system in their country. It identifies five crucial policy approaches that can be applied as appropriate to the context of the country – conducting situation analyses, sustaining a comprehensive strategy, engaging stakeholders, evaluating impacts on health policies and practices, and partnership participation. It also analyses, in turn, the individual functions and components within a system and identifies a series of tools that can be used for strengthening many of them.

If further analysis and research is thought to be relevant in the particular country looking to strengthen its health research system, this Opinion piece indicates some of the types of additional sources of information that are available. The Opinion piece also discusses aspects of the sometimes-controversial question of who should lead or steer attempts to strengthen NHRSs. Again, the context of the particular nation will be crucial in determining the most appropriate course to take, as emphasised by both Mansoori [32] and Atkinson et al. [33], but at least some involvement of the ministry of health is likely to be beneficial; additionally, sometimes, key individuals can play a crucial leadership role in strengthening the whole system or one component. In countries with a less developed tradition of conducting health research, partnerships with other countries and/or with international organisations can help lead the progress and learning for all partners. The valuable role that international organisations, such as WHO, can play in leading partnerships and cooperation to strengthen health research systems is being highlighted during the COVID-19 crisis.

Overall, therefore, the full WHO HEN report not only provides a detailed analysis of NHRS strengthening, it also provides a structure within which an even wider and ongoing literature can be considered. Additionally, it contains a perhaps more nuanced account, on which this paper builds, of some aspects of the literature around the issue of who should provide leadership in developing NHRSs and identifies the importance of ministry of health involvement.

## Data Availability

The full details of the papers included in the Health Evidence Network Evidence Synthesis are provided in that report, which is cited in this paper as reference [30]. The additional analysis of papers from *Health Research Policy and Systems* was based on the open access publications.

## Abbreviations

COHRED: Council on Health Research for Development
HARPS: Health Research Policy and Systems
HEN: Health Evidence Network
HRB: Health Research Board
NHRS: National Health Research System
NIHR: National Institute for Health Research
PAHO: Pan-American Health Organization
SDGs: Sustainable Development Goals
WAHO: West African Health Organization
